# Deep learning approach for early prediction of COVID-19 mortality using chest X-ray and electronic health records

**DOI:** 10.1186/s12859-023-05321-0

**Published:** 2023-05-09

**Authors:** Seung Min Baik, Kyung Sook Hong, Dong Jin Park

**Affiliations:** 1 Division of Critical Care Medicine, Department of Surgery, Ewha Womans University Mokdong Hospital, Ewha Womans University College of Medicine, Seoul, Korea; 2 Division of Critical Care Medicine, Department of Surgery, Ewha Womans University Seoul Hospital, Ewha Womans University College of Medicine, Seoul, Korea; 3 Department of Laboratory Medicine, Eunpyeong St. Mary’s Hospital, College of Medicine, The Catholic University of Korea, 1021, Tongil-ro, Eunpyeong-gu, Seoul, 03312 Korea

**Keywords:** COVID-19, Deep learning, Prediction model, Chest X-ray, Electronic health record

## Abstract

**Background:**

An artificial-intelligence (AI) model for predicting the prognosis or mortality of coronavirus disease 2019 (COVID-19) patients will allow efficient allocation of limited medical resources. We developed an early mortality prediction ensemble model for COVID-19 using AI models with initial chest X-ray and electronic health record (EHR) data.

**Results:**

We used convolutional neural network (CNN) models (Inception-ResNet-V2 and EfficientNet) for chest X-ray analysis and multilayer perceptron (MLP), Extreme Gradient Boosting (XGBoost), and random forest (RF) models for EHR data analysis. The Gradient-weighted Class Activation Mapping and Shapley Additive Explanations (SHAP) methods were used to determine the effects of these features on COVID-19. We developed an ensemble model (Area under the receiver operating characteristic curve of 0.8698) using a soft voting method with weight differences for CNN, XGBoost, MLP, and RF models. To resolve the data imbalance, we conducted F1-score optimization by adjusting the cutoff values to optimize the model performance (F1 score of 0.77).

**Conclusions:**

Our study is meaningful in that we developed an early mortality prediction model using only the initial chest X-ray and EHR data of COVID-19 patients. Early prediction of the clinical courses of patients is helpful for not only treatment but also bed management. Our results confirmed the performance improvement of the ensemble model achieved by combining AI models. Through the SHAP method, laboratory tests that indicate the factors affecting COVID-19 mortality were discovered, highlighting the importance of these tests in managing COVID-19 patients.

**Supplementary Information:**

The online version contains supplementary material available at 10.1186/s12859-023-05321-0.

## Background

Three years have passed since coronavirus disease 2019 (COVID-19) was discovered, but the spread of the virus has not ended globally [1]. The number of COVID-19 cases peaked in January 2022 and has been declining since then [2]. Although COVID-19 has a relatively low mortality rate [3], the number of infected people was so large at times that healthcare systems worldwide were in crisis owing to the large number of deaths.

COVID-19 research using artificial intelligence (AI), including deep learning (DL) and machine learning (ML), which have been studied extensively in the medical field, has been actively conducted (Table 1) [4–10]. AI is a concept that includes ML and DL. DL—a field of ML—is based on artificial neural networks, which are ML algorithms created by mimicking the principles and structures of human neural networks. DL models can be divided into deep neural networks (DNNs), convolutional neural networks (CNNs), and recurrent neural networks. DNNs are DL models that use multiple hidden layers and are useful for the analysis of high-dimensional data. Another DL class—the CNN—is used for the classification of image data, and there have been many studies on its usefulness [11, 12]. A CNN generates feature maps by applying convolution kernels to the input image. It proceeds with repeated convolution and pooling processes (feature extraction layer). Finally, the fully connected layer performs classification using extracted features [13]. Chest X-rays are easy to access and are often used for COVID-19 patients. Compared with other types of images, they are easy to collect and have a large amount of data; therefore, they have been widely used for DL targeting COVID-19 patients. In a previous study, the performance of a DL model using image data as a tool for diagnosing COVID-19 was acceptable (Table 1) [9]. However, in most studies, researchers developed classification models using normal and COVID-19 chest X-rays (Table 1) [10, 14].

**Table 1:** Artificial intelligence (AI) research related to COVID-19

References	Subject
Rahman et al. [4]	Diagnosis of COVID-19 using cough and breath sounds
Villavicencio et al. [5]	Early diagnosis of COVID-19 based on symptoms
Zhang et al. [6]	Classification of mild and severe cases of COVID-19 based on multivariate blood testing
Mahdavi et al. [7]	Prediction of COVID-19 mortality based on invasive and non-invasive clinical information
Yu et al. [8]	Prediction of mechanical ventilation and mortality for COVID-19 patients
Mohammad-Rahimi et al. [9]	Diagnosis of COVID-19 through X-ray and CT images
Bridge et al. [10]	Classification of COVID-19 and non-COVID-19 patients using CT images

*CT* computed tomography

For COVID-19, prognosis and mortality prediction are as important as the diagnosis. In January 2022, when COVID-19 incidence was at its highest, the world was overwhelmed by a shortage of medical capacity, increasing the number of deaths [2]. AI models for predicting the prognosis or mortality of COVID-19 patients will enable the efficient allocation of limited medical resources. In addition, useful information can be provided to the medical staff during treatment.

The contributions of this study are as follows. (1) We developed a medical AI model that utilizes initial chest X-ray and laboratory test data for early prediction of COVID-19 mortality. (2) We confirmed the prediction performance improvement of the ensemble model achieved by combining multiple AI models. (3) We identified specific clinical markers in COVID-19 mortality prediction. (4) We performed chest X-ray lesion visualization for COVID-19 mortality prediction. (5) We demonstrated the possibility of using electronic health record (EHR) data in DL.

## Results

A total of 304 COVID-19 patients were enrolled in this study, excluding two patients who died within 24 h of admission. The enrolled patients were categorized into a non-survival group (68 patients) and a survival group (236 patients). The mean age was 75.4 ± 10.86 years for the non-survival group and 66.0 ± 16.57 years for the survival group (*P* < 0.05). The proportions of patients with comorbidities, such as hypertension (*P* < 0.05), diabetes mellitus (*P* < 0.05), and kidney disease (*P* < 0.05), were higher in the non-survival group than in the survival group. The differences in the laboratory results between the two groups are presented in Additional file 1: Table S1.

### Performance of DL models using chest X-rays

**Fig. 1:**
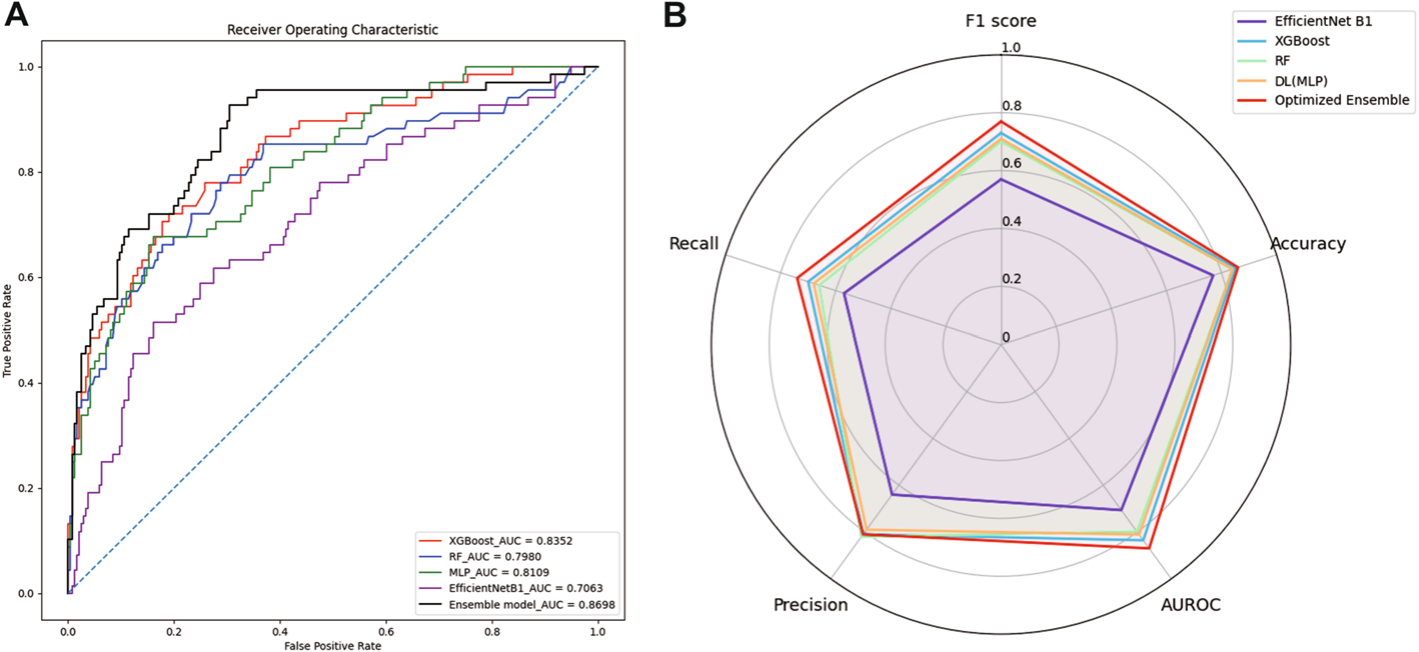
**A** AUROC of each model, including the ensemble model. **B** Rador plot for the performance of each model, including the ensemble model

Among the models, the overall mortality prediction performance was the best for EfficientNet B1, with an area under the receiver operating characteristic curve (AUROC) of 0.7063, accuracy of 0.77, precision of 0.64, recall of 0.57, and F1 score of 0.57, followed by EfficientNet B2 (AUROC of 0.6769, accuracy of 0.78, precision of 0.65, recall of 0.55, F1 score of 0.55), and Inception-ResNet-V2 (AUROC of 0.6166, accuracy of 0.76, precision of 0.50, recall of 0.50, F1 score of 0.46). In this study, EfficientNet B1 and EfficientNet B2 achieved better results than Inception-ResNet. Details are presented in Table 2 and Fig. 1.

**Table 2:** Performance of each model, including the ensemble model

Method	AUROC	Accuracy	Precision	Recall	F1 score
*Chest X-ray*					
Inception-ResNet-V2	0.6166	0.76	0.50	0.50	0.46
EfficientNet B2	0.6769	0.78	0.65	0.55	0.55
EfficientNet B1	0.7063	0.77	0.64	0.57	0.57
*EHR data*					
XGBoost	0.8352	0.85	0.81	0.70	0.73
RF	0.7980	0.84	0.82	0.66	0.70
DL (MLP)	0.8109	0.84	0.79	0.68	0.71
Ensemble model^a^	0.8698	0.84	0.86	0.66	0.69
Optimized ensemble model^b^	0.8698	0.86	0.81	0.74	0.77

*AUROC* area under the receiver operating characteristic curve, *EHR* electronic health record, *RF* random forest, *DL* deep learning, *MLP* multi-layer perceptron

^a^Ensemble model: Ensemble of EfficientNet B1, XGBoost, RF and DL (MLP)

^b^Ensemble model was optimized by F1 score (cutoff-value adjustment)

### Performance of DL (MLP) and ML models using EHR data

The results of the EHR comparisons between the survival and non-survival groups are presented in Additional file 1: Table S1.

Extreme Gradient Boosting (XGBoost) had the best mortality prediction performance (AUROC of 0.8352, accuracy of 0.85, precision of 0.81, recall of 0.70, F1 score of 0.73), followed by MLP (AUROC of 0.8109, accuracy of 0.84, precision of 0.79, recall of 0.68, F1 score of 0.71) and RF (AUROC of 0.7980, accuracy of 0.84, precision of 0.82, recall of 0.66, F1 score of 0.70). The performance of MLP was as good as that of the tree series, indicating the usefulness of MLP for hospital structured data (EHR) analysis. The performance of the prediction model using EHR data was better than that of the model using chest X-rays (Table 2, Fig. 1).

### Performance of ensemble model with DL (CNN, MLP) and ML (XGBoost, RF)

The performance of the ensemble model improved to 0.8698, with an accuracy of 0.84, a precision of 0.86, a recall of 0.66, and an F1 score of 0.69 (Table 2, Fig. 1A). The performance was apparently improved because the CNN model using images helped analyze the area for the prediction that could not be sufficiently explained with structured data alone. Although the AUROC of XGBoost among the models using EHR data was 0.8352 and that of EfficientNet B1 was approximately 0.7063 among the models using chest X-rays, the AUROC of our ensemble model was increased to 0.8698.

We performed F1-score optimization on the developed ensemble model because there was an imbalance between the numbers of surviving and non-surviving groups in the data. The F1 score is a classification metric that combines precision and recall. We performed F1-score optimization by adjusting the cutoff value to 0.35. As a result, the accuracy was increased from 0.84 to 0.86 and the F1 score was increased from 0.69 to 0.77 (Table 2, Fig. 1B), while the AUROC remained the same. The performance of the ensemble model with F1-score optimization was the best among the models developed (Fig. 1B).

The optimized ensemble model achieved an AUROC of 0.8698, an accuracy of 0.86, a precision of 0.81, a recall of 0.74, and an F1 score of 0.77, which were significant improvements.

### *Analysis of feature impact of EHR data *via* SHAP methods*

Although the DL model is unable to extract feature importance, we extracted the feature impact through the SHAP method for each model, including the DL model. We demonstrated the application of DL and ML for classifying COVID-19 mortality using EHR data.

**Fig. 2:**
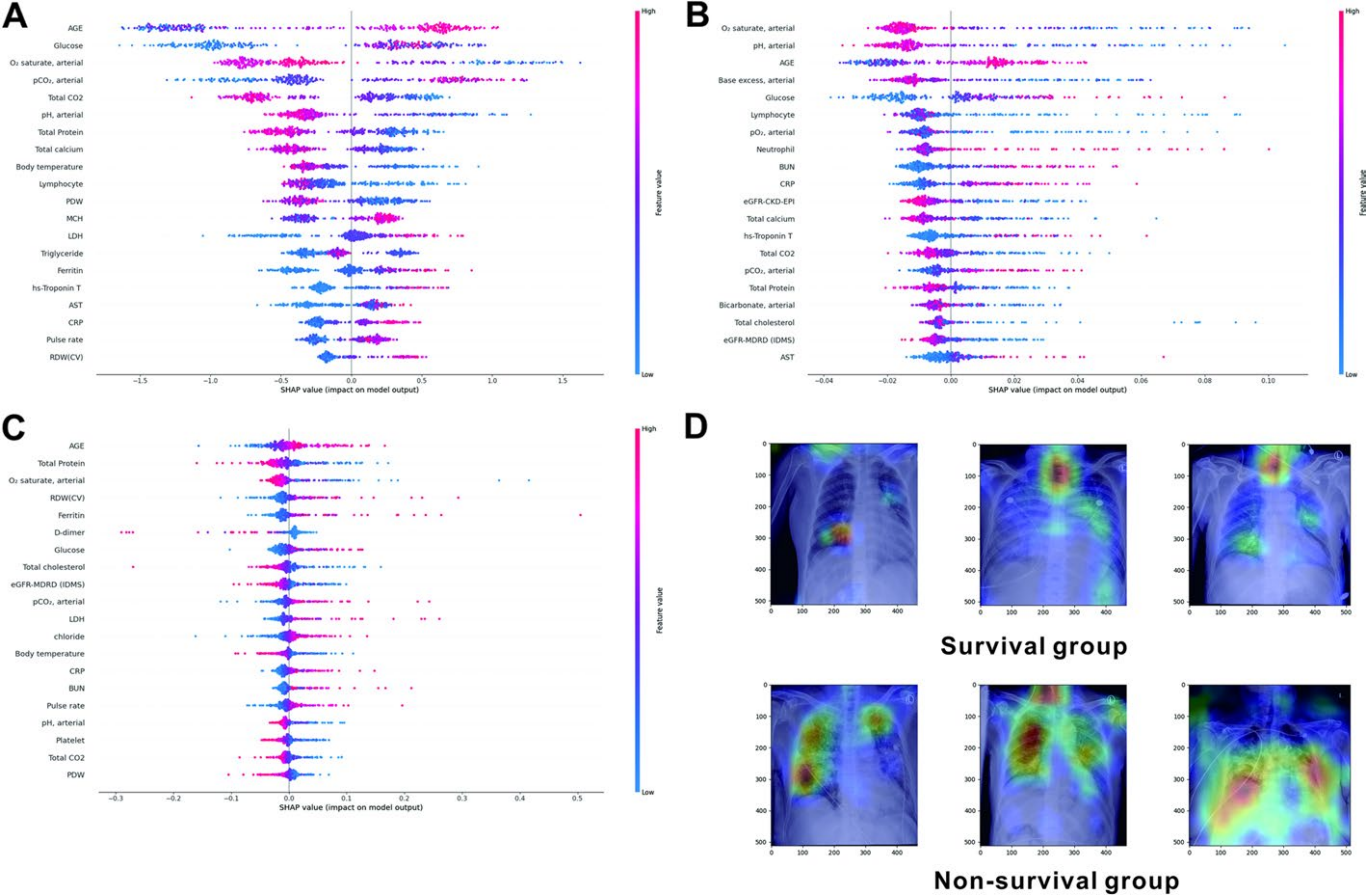
Shapley additive explanations (SHAP) method for feature impact and activation map visualization. **A** XGBoost, **B** Random forest, **C** Deep learning (Multilayer perceptron), **D** Activation map visualization for the survival and non-survival groups

The SHAP method provides a means of assessing the contributions of features to mortality. We employed it to obtain the feature impact of each ML (RF, XGBoost) and DL (MLP) model using EHR data, as shown in Fig. 2A–C. Here, blue indicates a negative correlation with mortality, and red indicates a positive correlation with death. The SHAP results for the models were as follows. For the XGBoost model, age had the largest feature impact, followed by serum glucose, O_2_ saturation, PaCO_2_, total CO_2_, and pH. For the RF model, O_2_ saturation had the largest feature impact, followed by pH, age, base excess, serum glucose, and lymphocyte (%). For the DL (MLP) model, age had the largest feature impact, followed by total protein, O_2_ saturation, red cell distribution width, ferritin, D-dimer, and serum glucose levels.

### Activation maps for survival and non-survival groups

Figure 2D shows the activation maps for the survival and non-survival groups. Regions highlighted in red indicate coarse localization mapping of regions recognized as important for COVID-19 mortality. There were visually significant differences between the Gradient weighted Class Activation Mapping (Grad-CAM) activation maps of the two groups. In the activation map of the non-surviving group, the highlighted part can be observed mainly in the lung than in the activation map of the survival group (Fig. 2D). Additionally, in the activation maps of the non-survival group, all regions of the lung (upper, middle, and lower lobes) were highlighted.

## Discussion

Our study is meaningful in that we developed an early mortality prediction model using only the initial chest X-ray and EHR data of COVID-19 patients. Early prediction of the clinical courses of patients is helpful for not only treatment but also bed management. Furthermore, chest X-rays and laboratory tests are readily available for patients with severe COVID-19 who are difficult to transport for advanced tests such as computed tomography. We developed an AI model using only chest X-rays and EHR data, which are routinely obtained for patients with severe COVID-19. We confirmed the performance improvement of the ensemble model achieved by blending AI models using materials with various characteristics, such as chest X-ray and EHR data. Through SHAP methods, laboratory tests that affect COVID-19 mortality were discovered, highlighting their importance in managing COVID-19 patients. All patients enrolled in our study had at least moderate severity of COVID-19, requiring a high-flow nasal cannula or advanced respiratory support, such as a mechanical ventilator. Accordingly, in the chest radiographs of both groups, significant lung lesions were observed in most cases. In mortality prediction, our CNN (EfficientNet B1) model using chest X-rays achieved an AUROC of 0.706. According to previous studies, the performance of the CNN model for diagnosing COVID-19 using normal chest X-rays and COVID-19 chest X-rays is relatively good [15, 16]. However, it is not easy to develop a mortality prediction model using a CNN for COVID-19 patients who have lung lesions in chest X-ray images [17, 18]. Therefore, we utilized EHR data, which are widely used in hospitals, to improve the performance of the prediction model. EHR data are largely structured, e.g., comorbidities, laboratory tests, and vital signs, and numerical. In general, DL models such as MLP are known to achieve good results for Big Data [19]. In this study, we used 23,712 datasets and applied the MLP model. In addition, to improve the prediction performance, an ML model (XGBoost, RF) with good classification performance was used [20]. In our study using EHR data, MLP exhibited a smaller AUROC than XGBoost but a larger AUROC than RF. Thus, the use of MLP can be considered in the analysis of structured hospital data.

We developed a model with improved performance using an ensemble of various AI models. In the ensemble process, optimal results were obtained under the following conditions: XGBoost, which achieved the highest AUROC, was assigned the largest weight; CNN (EfficientNet B1), which had the lowest AUROC, was assigned the second-largest weight; and MLP and RF were both assigned the smallest weight. In general, it is necessary to select AI models with various characteristics that perform well, and by assigning larger weights to models with better performance, models with improved performance can be developed. However, we obtained optimal results when we assigned large weights to the CNN model, which exhibited relatively poor performance. These results are presumed to be due to differences in the learning methods of the different AI models (CNN, MLP, ML) resulting from data with different characteristics (images and structured hospital data). Because most hospital data consist of images and EHR data (mostly structured data), similar to the data in our study, our ensemble technique is useful for developing a prediction model with good performance for respiratory diseases using hospital data.

An important point in the development of a mortality prediction model is that there is a data imbalance; i.e., there are less data for the non-survival group than for the survival group. Therefore, the F1 score is as important as the AUROC and accuracy for evaluating model performance. The F1 score is the harmonic mean of the precision and recall. In our data, there was a data imbalance between the two groups; thus, F1-score optimization (cutoff-value adjustment) was performed to improve the performance of the ensemble model (Table 2, Fig. 1B). For the development of mortality prediction models in the medical field, the F1-score optimization process performed in this study is worth considering.

Because the enrolled patients had moderate-to-severe disease, significant lesions were commonly observed on chest X-rays for both groups. Nevertheless, there was a clear difference in the activation map obtained using Grad-CAM between the two groups (Fig. 2D). Recently, several studies have been published on the application of Grad-CAM in various fields of medicine [21, 22]. Applying the activation map using Grad-CAM to COVID-19 patients is expected to help clinicians predict the patients’ hospital courses. In addition, we obtained information on the factors affecting COVID-19 mortality using SHAP methods, which have been recently introduced in the medical field [23, 24]. In the SHAP results of XGBoost, RF, and MLP, the O_2_ saturation and serum glucose level were commonly ranked high. Studies on the strong association between the worse clinical outcome of COVID-19 and hypoxemia have been conducted [25]. One study indicated that the survival rate of COVID-19 patients increased when the O_2_ saturation increased beyond 90.5% [26]. However, because COVID-19 is a respiratory disease, the importance of O_2_ saturation may not be a unique finding. Meanwhile, it is an interesting result that the serum glucose level ranks high for all three models in the SHAP results. Several studies on the association between the mortality of COVID-19 and diabetes mellitus have been reported. However, in our study, the serum glucose level alone exhibited importance. COVID-19 is easily transmitted by sepsis, and serum glucose levels must be maintained at an appropriate level in sepsis [27]. Therefore, the results of this study provide valuable evidence that the serum glucose level of COVID-19 patients should be properly maintained. Because the DL model is a black-box system, it is impossible to obtain information on the extent to which each parameter contributes to the performance of the prediction model. However, it is possible to investigate the feature impact for MLP using the SHAP method. With the development of AI technology in the medical field, the Grad-CAM and SHAP methods will help clinicians to evaluate patients.

The main limitation of our study was that it was conducted at a single institution with a small number of patients. Therefore, external validation was not performed on the developed model. However, to compensate for this, k-fold cross-validation was performed 10 times for chest X-ray images and 5 times for EHR data. We acknowledge that external validation is an important process in the development of AI models. In the future, we intend to collect data from multiple institutions for developing an improved prediction model.

## Conclusions

We developed a COVID-19 mortality early prediction model using only chest X-rays and EHR data, which are the most accessible data in hospitals, in which multiple AI models are combined to improve the prediction performance. Our model can help clinicians predict the clinical outcomes of COVID-19 patients as early as possible.

## Methods

### Patients and data collection

**Fig. 3:**
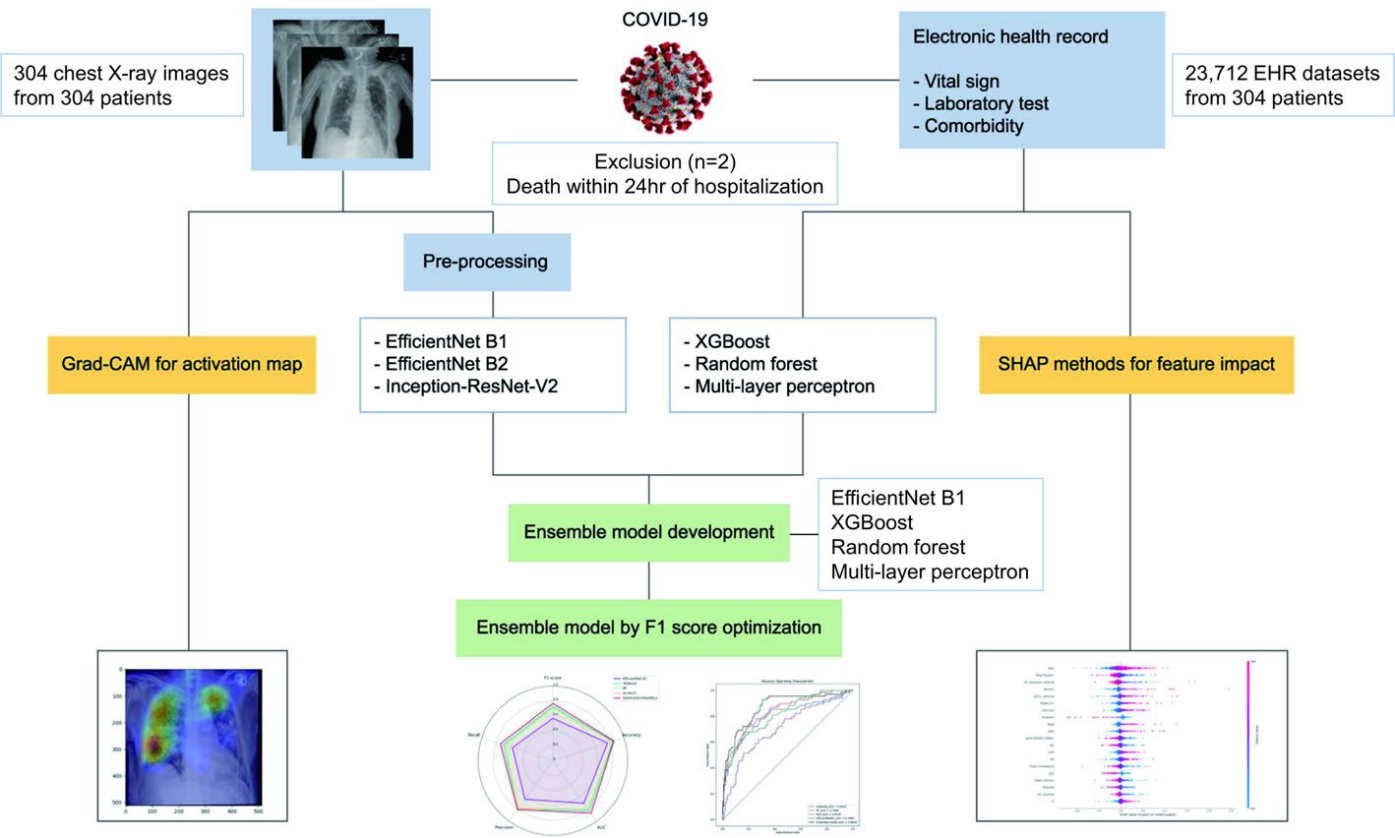
Flowchart of the development of the early prediction model for COVID-19 mortality

The overall process of the study is shown in Fig. 3.

This study included patients admitted to a tertiary hospital with a diagnosis of COVID-19 between September 2021 and May 2022. All participants required high-flow nasal cannula oxygen therapy or mechanical ventilation for respiratory assistance. All the patients underwent chest radiography and routine blood tests upon admission.

Because the objective of our study was to develop an early prediction model for mortality for COVID-19 patients, all the chest X-rays were limited to data acquired on the day of admission, and they were exported in the Digital Imaging and Communications in Medicine (DICOM) format.

EHR data, such as sex, age, medical history, and laboratory findings, were collected. The collected parameters are presented in Additional file 1: Table S1. All the collected EHR data and chest X-rays were anonymized.

### Data pre-processing

We collected initial chest X-rays in the DICOM format and converted them into Joint Photographic Experts Group (JPEG) files of 512 × 512 pixels. The best results were obtained by running the model with a batch size of 16 and image size of 512 × 512 pixels. In the case of an image size of 768 × 768 pixels, when the batch size was 16, it was overloaded, and when the batch size was 8, the performance was lower than that when the image size was 512 × 512 pixels, because of overfitting. Thus, we used 512 × 512 pixel JPEG files for DL, and it was possible to reduce the time consumption compared with using the original file directly in the CNN DL process.

Augmentation was performed on the converted chest X-ray files to develop the DL model with improved performance. ImageDataGenerator was used for pre-processing in the TensorFlow framework. The augmentation data were used for model training, whereas data without augmentation were used for model validation.

We acquired 23,712 EHR datasets, and the missing value of 1859 (7.8%) was pre-processed as the median value for the DL model. The EHR data used in this study consisted of 78 parameters, including sex and age, comorbidity, arterial blood gas analysis results, vital signs, and laboratory results of 61 tests (Additional file 1: Table S1). During the EHR data pre-processing step, the range of the parameters was standardized and scaled using the “scikit-learn” Python library.

### DL (CNN) model development for chest X-ray image analysis

In the case of image data (chest X-ray analysis), we utilized CNN models, including EfficientNet B1, EfficientNet B2, and Inception-ResNet-V2. EfficientNet is an optimized model that was developed through multiple experiments and consists of reinforcement-learning structures [28]. The EfficientNet and Inception-ResNet-v2 models exhibit excellent performance for image classification [29]. In DL model training, increasing the number of epochs improves the performance; however, if the number of epochs is excessive, the performance deteriorates owing to overfitting. Because optimization is performed at the best validation loss value, it was performed using the early stopping technique during training to prevent an excessive increase in the number of epochs. Finally, the number of epochs was set as 40, and early stopping (patience = 8) was used to stop learning if the validation loss did not improve during the 8 additional epochs. Because this study was conducted using initial chest X-rays of 304 patients, augmentation and k-fold validation were used to improve the model performance. K-fold cross-validation has a significant advantage in that all data can be utilized. In this study, image pre-processing was performed using the ImageDataGenerator library to learn image data in TensorFlow framework, and the validation loss and F1 score were used as evaluation indices. Image data classification was performed using the Inception-ResNet-V2, EfficientNet B1, and EfficientNet B2 models, and the number of epochs was set as 40. The evaluation index for the CNN model was the validation loss, and the early stopping technique was used. The validation loss, which was the evaluation index for the developed model, decreased as the number of epochs increased and was optimized for the epoch with the smallest validation loss. If the number of epochs increases, even if the first smallest validation loss occurs, the early stopping technique uses the option of patience to execute additional epochs, and if a lower validation loss occurs, training is continued. In this study, the patience of 8 was used, and among the three CNN models, EfficientNet B1 achieved the best AUROC.

### Development of DL (MLP) and ML models for EHR data analysis

In the case of EHR data, we selected DL models such as MLP and ML models such as XGBoost and RF. MLP is a class of DNN that consists of at least three layers: the input, hidden, and output layers [30]. Because the EHR data mainly comprise quantitative results, MLP was used. Tree-based ML, such as XGBoost and RF, exhibits excellent classification performance [31]. ML and DL analyses were performed using 23,712 datasets. K-fold cross-validation (n_split:5) was applied to all the datasets to prevent data loss. We performed DL (MLP) in addition to ML for classification using EHR data with a data imbalance. With regard to MLP, the best performance was achieved when one hidden layer of MLP was used. When two or more hidden layers were stacked, the performance was poor because of overfitting.

### Ensemble model development for performance improvement

The ensemble technique combines two or more related but different analytical models, and the results are blended into an ensemble spread to improve the prediction performance [32, 33]. We developed an ensemble model by combining the EfficientNet B1 model using chest X-rays with XGBoost, MLP, and RF using EHR data. Ensemble techniques can be divided into three main types: hard voting, soft voting, and weighted voting [34, 35]. In this study, the models were assembled using the blending technique of weighted voting. The models selected for the ensemble were EfficientNet B1, which exhibited the best performance among the CNN models, and MLP, XGBoost, and RF for EHR data analysis. Using these four models, we developed an ensemble model by assigning weights (0.3 for EfficientNet B1, 0.4 for XGBoost, and 0.15 for MLP and RF). Therefore, we used the ensemble technique with DL (CNN) of chest X-rays and DL (MLP) and ML (XGBoost and RF) of EHR data to improve the COVID-19 mortality prediction performance.

### F1-score optimization of ensemble model

The performance of the models was evaluated according to the AUROC, accuracy, precision, recall, and F1 score. The F1 score is the harmonic average of the precision and recall. In the analysis with data imbalance, both the accuracy and the F1 score were used to evaluate the classification performance. We optimized the F1 score with a cutoff adjustment (0.35) to develop a model that could predict both classes in a balanced manner.

### Stratified k-fold cross-validation

We performed k-fold validation using both chest X-rays and EHR data. The cross-validation method used in the sensitivity analysis of k-fold cross-validation in prediction error estimation was used to generate more general models for more realistic profiles [36–38]. In the CNN models (EfficientNet B1, EfficientNet B2, and Inception-ResNet-V2) for chest X-ray analysis, the following k-fold validation (n_split:10) was performed to maximize the image data utilization and avoid data loss: training with 90% data, validation with 10% data, and repeating this process 10 times. For the EHR data analysis, k-fold validation (n_split:5) was performed to avoid data loss.

### Activation map visualization for chest X-ray and SHAP method for EHR data

We implemented the Grad-CAM technique in a pipeline for the visual explanation of chest X-rays for COVID-19 mortality prediction. The Grad-CAM technique utilized for the visual explanation of CNN-based models creates a coarse localization map that highlights important areas of the image [39].

In addition, EHR data analysis using the Shapley Additive Explanations (SHAP) method was performed used to evaluate the impact of the features on COVID-19 mortality. The “Shapley” value is a concept in game theory that indicates the contributions of different features to a particular outcome. SHAP values were obtained using Deep Learning Important Features (DeepLIFT) by propagating activation differences [40]. DeepLIFT for the SHAP value of DL (MLP) is a method for decomposing the output prediction of a neural network for a specific input by backpropagating all features to extract the contribution of all neurons in the network. We used the SHAP method to investigate the features that contributed to COVID-19 mortality in our EHR data for ML (RF, XGBoost) and DL (MLP).

## Supplementary Information

**Additional file 1. Table S1.** Clinical characteristics and laboratory results of COVID-19 patients.

## Data Availability

The datasets used for analyses in this study are available from the corresponding author upon reasonable request.

## Abbreviations

COVID-19: Coronavirus disease 2019
AI: Artificial intelligence
DL: Deep learning
ML: Machine learning
DNN: Deep neural networks
CNN: Convolutional neural networks
EHR: Electronic health record
DICOM: Digital Imaging and Communications in Medicine
JPEG: Joint Photographic Experts Group
MLP: Multilayer perceptron
XGBoost: Extreme Gradient Boosting
RF: Random forest
AUROC: Area under the receiver operating characteristic curve
SHAP: Shapley additive explanations
Grad-CAM: Gradient-weighted class activation mapping
DeepLIFT: Deep learning important features
