# Supplementary material for: Deep learning approach for early prediction of COVID-19 mortality using chest X-ray and electronic health records
Source: BMC Bioinformatics. 2023 May 9;24:190. doi: 10.1186/s12859-023-05321-0 (PMC10169101; doi:10.1186/s12859-023-05321-0)
Supplement: Supplementary file 1 — Additional file 1. Table S1. Clinical characteristics and laboratory results of COVID-19 patients. [file 12859_2023_5321_MOESM1_ESM.docx]

**Table S1. Clinical characteristics and laboratory results of COVID-19 patients**

| **Variable** | **Non-survival (n = 68)** | **Survival (n =236)** | ***P* value** |
| --- | --- | --- | --- |
| Sex, male (%) | 35 (51.5) | 120 (50.8) | 0.901 |
| Age (years) | 75.4 ± 10.86 | 66.0 ± 16.57 | < 0.05 |
| Systolic blood pressure (mmHg) | 131.0 ± 25.70 | 132.3 ± 22.08 | 0.704 |
| Diastolic blood pressure (mmHg) | 73.7 ± 14.36 | 79.4 ± 15.99 | < 0.05 |
| Pulse rate (bpm) | 85.9 ± 23.16 | 84.9 ± 18.87 | 0.724 |
| Respiratory rate (bpm) | 24.2 ± 14.90 | 21.9 ± 9.75 | 0.242 |
| Pulse pressure (mmHg) | 57.3 ± 20.06 | 52.9 ± 17.28 | 0.072 |
| Mean blood pressure (mmHg) | 92.8 ± 16.37 | 97.0 ± | 0.058 |
| Comorbidities |  |  |  |
| Hypertension | 46 (67.6) | 104 (44.1) | < 0.05 |
| Diabetes mellitus | 32 (47.1) | 63 (26.7) | < 0.05 |
| Heart disease | 12 (17.6) | 27 (11.4) | 0.178 |
| Lung disease | 4 (5.9) | 22 (9.3) | 0.372 |
| Liver disease | 1 (1.5) | 11 (4.7) | 0.234 |
| Kidney disease | 10 (14.7) | 10 (4.2) | < 0.05 |
| Brain disease | 9 (13.2) | 47 (19.9) | 0.211 |
| Malignant disease | 9 (13.2) | 38 (16.1) | 0.565 |
| White blood cell count (×10^3^/uL) | 11.20 ± 7.488 | 8.25 ± 4.828 | < 0.05 |
| Red blood cell count (×10^6^/uL) | 3.74 ± 0.775 | 4.03 ± 0.941 | < 0.05 |
| Hemoglobin (g/dL) | 11.30 ± 2.268 | 12.33 ± 5.580 | 0.142 |
| Hematocrit (%) | 32.9 ± 6.25 | 34.7 ± 6.45 | < 0.05 |
| Mean corpuscular volume (fL) | 88.7 ± 5.99 | 87.6 ± 7.94 | 0.283 |
| Mean corpuscular hemoglobin (pg) | 30.4 ± 2.37 | 30.1 ± 2.96 | 0.370 |
| Mean corpuscular hemoglobin concentration (%) | 34.3 ± 1.32 | 34.7 ± 6.73 | 0.565 |
| Red cell distribution width | 13.9 ± 1.86 | 13.4 ± 1.76 | < 0.05 |
| Platelet (×10^3^/uL) | 171.6 ± 87.62 | 215.3 ± 97.04 | < 0.05 |
| Plateletcrit (%) | 0.18 ± 0.085 | 0.72 ± 7.486 | 0.564 |
| Mean Platelet Volume (fL) | 10.7 ± 0.92 | 10.7 ± 5.37 | 0.982 |
| Platelet volume distribution width (fL) | 12.3 ± 2.51 | 11.9 ± 5.96 | 0.639 |
| Automated absolute neutrophil count (×10^3^/uL) | 10.16 ± 7.284 | 6.70 ± 4.601 | < 0.05 |
| Automated neutrophil (%) | 88.3 ± 7.45 | 80.9 ± 12.38 | < 0.05 |
| Automated lymphocyte (%) | 7.4 ± 5.63 | 12.4 ± 8.25 | < 0.05 |
| Automated monocyte (%) | 3.9 ± 2.71 | 6.0 ± 4.48 | < 0.05 |
| Automated eosinophil (%) | 0.2 ± 0.65 | 0.8 ± 5.06 | 0.349 |
| Automated basophil (%) | 0.2 ± 0.25 | 0.2 ± 0.18 | 0.948 |
| Activated Partial Thromboplastin Time (sec) | 31.16 ± 6.490 | 32.82 ± 10.104 | 0.233 |
| Prothrombin Time (sec) | 14.7 ± 3.190 | 14.2 ± 11.76 | 0.793 |
| Prothrombin Time (%) | 74.2 ± 21.91 | 83.0 ± 18.29 | < 0.05 |
| Prothrombin Time (INR) | 1.247 ± 0.2775 | 1.845 ± 8.1027 | 0.614 |
| Total calcium (mg/dL) | 7.79 ± 0.748 | 8.29 ± 1.097 | < 0.05 |
| Phosphorus (mg/dL) | 3.50 ± 1.187 | 3.05 ± 0.975 | < 0.05 |
| Glucose (mg/dL) | 214.2 ± 74.69 | 165.0 ± 71.16 | < 0.05 |
| Blood urea nitrogen (mg/dL) | 38.6 ± 26.05 | 21.8 ± 18.05 | < 0.05 |
| Creatinine (mg/dL) | 1.69 ± 1.541 | 1.04 ± 1.175 | < 0.05 |
| eGFR-MDRD -IDMS (mL/min/1.73 m2) | 60.1 ± 37.63 | 94.1 ± 47.02 | < 0.05 |
| eGFR-CKD-EPI (mL/min/1.73 m2) | 55.3 ± 31.41 | 83.1 ± 30.00 | < 0.05 |
| Triglyceride (mg/dL) | 129.8 ± 79.26 | 118.1 ± 67.58 | 0.270 |
| Total cholesterol (mg/dL) | 110.2 ± 44.29 | 133.2 ± 37.63 | < 0.05 |
| Total protein (g/dL) | 5.71 ± 0.737 | 6.15 ± 0.628 | < 0.05 |
| Albumin (g/dL) | 3.01 ± 0.493 | 3.36 ± 0.556 | < 0.05 |
| Aspartate aminotransferase (IU/L) | 87.5 ± 190.30 | 76.6 ± 383.44 | 0.821 |
| Alanine aminotransferase (IU/L) | 40.1 ± 55.64 | 50.2 ± 202.96 | 0.689 |
| Alkaline phosphatase (IU/L) | 103.5 ± 65.66 | 90.5 ± 53.36 | 0.140 |
| Total bilirubin (mg/dL) | 0.60 ± 0.520 | 1.85 ± 20.699 | 0.630 |
| Sodium (mmol/L) | 137.1 ± 5.22 | 136.6 ± 9.82 | 0.686 |
| Potassium (mmol/L) | 4.4 ± 0.64 | 4.2 ± 0.66 | < 0.05 |
| Chloride (mmol/L) | 104.9 ± 5.87 | 103.5 ± 8.52 | 0.201 |
| Total carbon dioxide (mmol/L) | 17.3 ± 4.01 | 21.0 ± 19.96 | 0.124 |
| C-reactive protein (mg/dL) | 14.63 ± 7.747 | 10.62 ± 19.405 | 0.103 |
| Creatine kinase (IU/L) | 412.3 ± 640.56 | 270.6 ± 1074.37 | 0.405 |
| Creatine kinase-MB (ng/mL) | 5.28 ± 6.043 | 3.05 ± 7.006 | 0.055 |
| High sensitivity-Troponin T (ng/mL) | 0.152 ± 0.4499 | 0.035 ± 0.1086 | 0.077 |
| Procalcitonin (ng/mL) | 7.65 ± 15.764 | 1.87 ± 7.553 | < 0.05 |
| Lactate dehydrogenase (IU/L) | 656.4 ± 616.26 | 424.0 ± 285.09 | < 0.05 |
| N-terminal pro-B-type natriuretic peptide (pg/mL) | 5264.9 ± 8098.74 | 1398.7 ± 3850.11 | < 0.05 |
| Uric acid (mg/dL) | 5.41 ± 2.612 | 4.00 ± 2.153 | < 0.05 |
| Ferritin (ng/mL) | 1571.6 ± 1467.73 | 1007.5 ± 948.75 | < 0.05 |
| Fibrinogen (mg/dL) | 446.2 ± 156.61 | 436.7 ± 154.99 | 0.711 |
| Fibrinogen degradation production (ug/mL) | 20.81 ± 27.394 | 9.69 ± 17.020 | < 0.05 |
| D-dimer (mg/L) | 4.14 ± 5.936 | 2.46 ± 3.838 | 0.104 |
| Arterial blood gas analysis |  |  |  |
| pH | 7.375 ± 0.1155 | 7.434 ± 0.2256 | < 0.05 |
| PaCO_2_ (mmHg) | 35.7 ± 11.91 | 33.0 ± 7.02 | 0.086 |
| PaO_2_ (mmHg) | 85.4 ± 40.40 | 102.7 ± 57.83 | < 0.05 |
| Bicarbonate (mmol/L) | 20.3 ± 4.31 | 22.5 ± 4.01 | < 0.05 |
| Base excess (mmol/L) | -4.5 ± 5.40 | -1.1 ± 4.38 | < 0.05 |
| CO_2_ content (mmol/L) | 21.4 ± 4.41 | 23.5 ± 4.15 | < 0.05 |
| O_2_ saturation (%) | 91.2 ± 12.65 | 96.1 ± 2.98 | < 0.05 |
| Lactate (mg/dL) | 20.6 ± 21.23 | 15.7 ± 10.68 | 0.116 |
